# *Campylobacter jejuni* CsrA Regulates Metabolic and Virulence Associated Proteins and Is Necessary for Mouse Colonization

**DOI:** 10.1371/journal.pone.0156932

**Published:** 2016-06-03

**Authors:** Joshua A. Fields, Jiaqi Li, Connor J. Gulbronson, David R. Hendrixson, Stuart A. Thompson

**Affiliations:** 1 Department of Medicine, Division of Infectious Diseases, Augusta University, Augusta, GA, 30912, United States of America; 2 Department of Natural Sciences, Georgia Military College - Augusta, Augusta, GA, 30907, United States of America; 3 Department of Microbiology, University of Texas Southwestern Medical Center, Dallas, TX, 75390, United States of America; East Carolina University School of Medicine, UNITED STATES

## Abstract

*Campylobacter jejuni* infection is a leading bacterial cause of gastroenteritis and a common antecedent leading to Gullian-Barré syndrome. Our previous data suggested that the RNA-binding protein CsrA plays an important role in regulating several important phenotypes including motility, biofilm formation, and oxidative stress resistance. In this study, we compared the proteomes of wild type, *csrA* mutant, and complemented *csrA* mutant *C*. *jejuni* strains in an effort to elucidate the mechanisms by which CsrA affects virulence phenotypes. The putative CsrA regulon was more pronounced at stationary phase (111 regulated proteins) than at mid-log phase (25 regulated proteins). Proteins displaying altered expression in the *csrA* mutant included diverse metabolic functions, with roles in amino acid metabolism, TCA cycle, acetate metabolism, and various other cell processes, as well as pathogenesis-associated characteristics such as motility, chemotaxis, oxidative stress resistance, and fibronectin binding. The *csrA* mutant strain also showed altered autoagglutination kinetics when compared to the wild type. CsrA specifically bound the 5’ end of *flaA* mRNA, and we demonstrated that CsrA is a growth-phase dependent repressor of FlaA expression. Finally, the *csrA* mutant exhibited reduced ability to colonize in a mouse model when in competition with the wild type, further underscoring the role of CsrA in *C*. *jejuni* colonization and pathogenesis.

## Introduction

*Campylobacter jejuni* is a leading bacterial cause of gastroenteritis throughout the world. As a pathogen of significant public health importance, *C*. *jejuni* has been extensively studied; however, our understanding of the exact mechanisms by which it causes disease remain incomplete. Although most frequently associated with poultry due to a commensal relationship with avian species and the frequent occurrence of disease following the ingestion of undercooked chicken, *C*. *jejuni* inhabits a broad range of habitats, both enteric and environmental, requiring it to regulate gene expression accordingly. Analysis of the *C*. *jejuni* genome indicates that gene regulation is limited to a fraction of the regulatory elements found in other bacteria [1]. However, *C*. *jejuni* has a homolog of the *E*. *coli* post-transcriptional regulator, CsrA. In *E*. *coli*, CsrA regulates the translation of target proteins by binding to sequences containing the motif, ANGGA, which is often found overlapping or adjacent to the ribosome binding site (RBS) of target mRNAs [2]. This action of CsrA upon its target is capable of repressing translation by blocking the availability of the RBS for the ribosome as well as either decreasing or increasing mRNA stability [3, 4]. We previously reported that mutation of *C*. *jejuni csrA* disrupts a number of important phenotypes [5]. In the absence of CsrA, *C*. *jejuni* cells display reduced motility, decreased ability to accumulate biofilms when grown in static culture, an increased sensitivity to oxidative stresses, and defective adherence to human intestinal epithelial cells *in vitro*. The *csrA* mutant was also hyperinvasive in the same intestinal epithelial cell model.

The majority of bacteria in nature exist not as planktonic cells, but as biofilm communities of one or more species held together by an extracellular polymeric substance (EPS)[6] and/or DNA [7]. *Campylobacter*-containing biofilms occur in numerous niches in nature, including poultry houses and various environmental water locations [8, 9]. Furthermore, biofilm formation appears important for *C*. *jejuni* survival in slaughterhouses and on contaminated meats [10]. These mono- or multi-species biofilms may contribute to survival of the fastidious and oxygen-sensitive *Campylobacter* in nutrient-poor, aerobic environments, and may play a role in the transmission of *C*. *jejuni* both within poultry farms, and to humans from the environment. Biofilms can also form on mucosal surfaces during infections; biofilms allow the bacteria within them to become highly resistant to elimination by the host immune system or by antibiotic therapy. *Campylobacter* biofilms form readily on *ex vivo* primary human intestinal tissue and thus may be important during human infection [11]. Although the formation of *C*. *jejuni* biofilms is not fully understood, as with many other bacteria they contain DNA [7, 12] and polysaccharide [13].

In this study, we used proteomics to identify both direct and indirect targets of CsrA, in an effort to define the *C*. *jejuni* CsrA regulon and elucidate molecular mechanisms for the phenotypes observed in the *csrA* mutant. We found that the effect of CsrA on the expression of whole cell proteins was more profound at stationary phase growth as compared to mid-log. While the expression of 25 proteins was altered in *csrA* mutant cells grown to mid-log phase, when grown to stationary phase, 111 proteins were differentially expressed in the absence of CsrA. We also demonstrated a direct role for the growth-phase-dependent regulation of FlaA (flagellin) expression via specific binding of CsrA to the 5’ end of *flaA* mRNA. The *csrA* mutant also displayed altered autoagglutination kinetics in comparison to the wild type strain, and decreased ability to experimentally colonize mice.

## Materials and Methods

### Bacterial strains and routine growth conditions

All bacterial strains used in this study are listed in Table 1. *C*. *jejuni* strain 81–176 [14], it isogenic *csrA* mutant, and complemented mutant strain *csrA*/pJF11 [5] were stored at -80°C in Mueller Hinton (MH) broth (0.2% beef extract, 1.75% acid digest of casein, 0.15% starch) containing 20% (v:v) glycerol and grown on MH agar at 42°C in a tri-gas incubator (85% N_2_, 10% CO_2_, 5% O_2_) or in microaerophilic atmospheres generated within Mitsubishi AnaeroPack jars using AnaeroPouch microaerophilic gas generator sachets (Remel). For some experiments, *C*. *jejuni* strains were grown in MH biphasic cultures. To select for *C*. *jejuni* mutant and complemented strains, chloramphenicol (30 μg/ml) and kanamycin (30 μg/ml) were used. *E*. *coli* strains were stored at -80°C in Luria-Bertani (LB) broth (1% tryptone, 0.5% yeast extract, 1% NaCl) containing 20% (v:v) glycerol, and routine growth was carried out at 37°C on LB agar or in LB broth with shaking. When appropriate, *E*. *coli* strains were selected in LB medium using ampicillin (100 μg/ml) or kanamycin (50 μg/ml).

**Table 1:** Bacterial strains, plasmids, and primers used in this study.

Strain or Plasmid	Description	Resistance	Source or Reference
**Strain**			
***Campylobacter jejuni***			
81–176	Wild type		[14]
81–176*ΔcsrA*	*C*. *jejuni csrA* mutant	Cm	[5]
81–176 *ΔcsrA/*pJF11	complemented *csrA* mutant	Cm, Kan	[5]
***Escherichia coli***			
JM109	Cloning host		Promega
BL21(DE3)	Protein expression strain		Promega
**Plasmids**			
pCRII-TOPO	Cloning vector	Amp, Kan	Invitrogen
pET-20b(+)	Protein expression vector	Amp	Novagen
pJAF50	*csrA* cloned into pCRII-TOPO	Amp, Kan	This study
pJAF51	*csrA* cloned into pET-20b(+)	Amp	This study
**Primers**			
JAF*csrA*NdeI	GGCATATGTTAATATTATCAAGAAAAGAAAATG		This study
JAF*csrA*XhoI	GGCTCGAGTTTGATTAGTTTTTTGCTTAAGTC		This study
JL107	TAATACGACTCACTATAGGGTATAAATTTTTAAAAAAAAG		This study
JL108	TTTAAATCCTTTTAAATAATTTC		This study
*phoB*T7-F	TAATACGACTCACTATAGGGCATTAATGATCGCAACCTATTTATTACAACAGGGCAAATCAT		[15]
*phoB*T7-R	CATGATTTGCCCTGTTGTAATAAATAGGTTGCGATCATTAATGCCCCTATAGTGAGTCGTATTA		[15]

### Two dimensional protein gels

Proteomics experiments were performed as previously described [16] employing differential in-gel electrophoresis (DIGE) methodology and a GE Biosystems semi-automated workstation. Briefly, wild type, *csrA* mutant, and complemented *C*. *jejuni* cells were grown to mid-log (OD_600_ = 0.5) or stationary phase (OD_600_ = 1.0) in shaking cultures at 42°C. The cultures where then harvested on ice and RNA and protein synthesis were halted as described [16]. Cells were collected by centrifugation, and cell lysates prepared as described [16]. The protein concentrations of the lysates were determined using the BCA assay kit (Pierce). Next, 25 μg of protein each from wild type, *csrA* mutant, and complemented strains were labeled in the dark with 1 μl Cy2, Cy3, or Cy5 dye conjugates respectively according to the manufacturer’s instructions (GE Biosystems). The labeled proteins were mixed with the corresponding unlabeled proteins in equal amounts (to total 50 μg of each protein sample), subjected to isoelectric focusing (IEF) (IPGPhor strips, range 3–10, non-linear), and then separated by size on 12% SDS polyacrylamide gels.

Following SDS-PAGE, the gel was scanned on a Typhoon fluorescent flatbed scanner (GE Biosystems) at the appropriate wavelengths for Cy2, Cy3, and Cy5 [16]. These images were then overlaid and proteins exhibiting altered expression in the *csrA* mutant compared to either the wild type or complemented strains were identified using Decyder Differential In-Gel Analysis (DIA) software (version 4.0, GE Biosystems). Proteins of interest were excised, digested with trypsin (Invitrogen), and analyzed by using MALDI-ToF/ToF spectrometry (Applied Biosystems). Protein identifications were a result of searching protein databases with tryptic fingerprint data and primary amino acid sequences of fragmented peptides following MS/MS. Proteomics experiments were performed on three biological replicates. Proteins were mapped to functional categories using the KEGG database [17] or according to known functions (e.g. motility/chemotaxis) to emphasize their roles in *C*. *jejuni* biology.

### Purification of recombinant C-terminally His-tagged CsrA

Recombinant *C*. *jejuni* CsrA containing a C-terminal His_6_ tag was purified by affinity chromatography. PCR primers and plasmids used in this work are listed in Table 1. Primers JAF*csrA*NdeI and JAF*csrA*XhoI (Table 1) were used in PCR reactions to amplify the *csrA* coding sequence (lacking the stop codon) from 81–176 genomic DNA. The resulting amplicon was then cloned into the TA-cloning vector pCRII-TOPO (Invitrogen) to generate the plasmid pJAF50. The insert was excised by restriction endonuclease digestion with the enzymes *XhoI* and *NdeI*, gel purified and subcloned into the *E*. *coli* expression vector pET-20b(+) (Novagen) to yield plasmid pJAF51. Plasmid pJAF51 was then transformed into *E*. *coli* expression strain BL21(DE3) for use in overexpression and purification. This strain was grown in two liters of LB broth supplemented with ampicillin (50 μg/ml) to mid-log (OD_600_ = 0.5), then was induced to express recombinant His-tagged CsrA by the addition of 1 mM IPTG for three hours. After the induction period, the culture was pelleted and washed with STE buffer (0.1 M NaCl, 10 mM Tris-HCL, pH 8, 1 mM EDTA, pH 8) and resuspended in lysis buffer (50 mM Tris, pH 7.8, 2 mM β-mercaptoethanol, 5% glycerol, 2 mM EDTA, pH 8) and homogenized. The lysate was then further incubated with an additional 4 ml of lysis buffer for 30 minutes followed by sonication on ice ten times at ten second intervals punctuated by 10 second pauses to prevent overheating of the lysate. The sonicated lysate was centrifuged at 12,000 rpm, retaining the supernatant for further purification steps. The supernatant was fractionated by incubation for 1 hour in the presence of ammonium sulfate (0.56 g/ml) and centrifuged as above. The pellet was resuspended in buffer CJA (125 mM Tris, pH 7.5, 5 mM β-mercaptoethanol, 2.5% glycerol, 2 mM EDTA, pH 8) and dialyzed overnight at 4°C against buffer CJA. Imidazole (50 mM) was added to the dialyzed lysate for binding to a Nickel-Sepharose column. After washing, bound protein was eluted by the addition of buffer CJA containing 300 mM imidazole, and fractions were collected for further analysis. Fractions containing purified His-tagged CsrA were then pooled and dialyzed against a storage buffer (50 mM Tris, pH 8, 50% glycerol, 2 mM EDTA, 100 mM NaCl) to concentrate the sample. Purified and concentrated CsrA-His_6_ aliquots were stored at -20°C for future experiments. Rabbit polyclonal santiserum to CsrA-His_6_ was prepared by a commercial vendor (Cocalico Biologicals).

### FlaA expression during growth curve

To test for CsrA- and growth-phase-dependent expression of FlaA, we grew WT and *csrA* mutant *C*. *jejuni* as follows. Strains 81–176 and the *csrA* mutant were grown overnight in MH biphasic cultures at 42°C. Overnight cultures were then diluted into fresh MH broth to an initial OD_600_ = 0.1, and incubated at 37°C. Samples were removed at 5, 6, 8, and 10 hours of incubation and subjected to western blots using FlaA-specific polyclonal antiserum. Samples for western blots were normalized for protein content, determined by BCA assay.

### Electrophoretic Mobility Shift Assay (EMSA)

EMSA experiments were performed essentially as described [18]. DNA templates to be used for *in vitro* RNA transcription were generated by using PCR and primers containing T7 promoters (Table 1), designed to correspond to the region encompassing the 5’ end of mRNA for either *C*. *jejuni flaA* or *E*. *coli phoB* (as a CsrA-non-binding control)[15]. RNA was then synthesized from these purified PCR products using the MEGAscript kit (Ambion). The transcripts were purified via phenol:chloroform extraction, followed by ethanol precipitation. Purified RNA was resuspended in TE buffer, heated to 85°C, and allowed to cool to room temperature on the bench. The transcripts were then incubated with concentrations of purified, recombinant CsrA-His_6_ ranging from 0–300 nM, in the presence of yeast RNA in 10 μl reactions (10 mM Tris-HCl, pH 7.5, 10 mM MgCl_2_, 100 mM KCl, 32.5 ng total yeast RNA, 20 mM DTT, 7.5% glycerol) and 4 U of RNase inhibitor (Ambion) for 30 minutes at 37°C. The reactions were resolved on 15% native polyacrylamide gels and scanned on a Bio-Rad phosphorimager. The dissociation constant (Kd) was calculated as described [19].

### Autoagglutination assay

Autoagglutination of *C*. *jejuni* wild type, *csrA* mutant and complemented *csrA* mutant strains was performed as previously described [20]. Overnight, liquid cultures of *C*. *jejuni* were diluted to an OD_600_ of 1.0 in phosphate buffered saline (PBS), distributed into 2 ml aliquots and incubated at room temperature. The OD_600_ of the top 1 ml of the culture was measured at 2, 4, 6, and 24 hours to determine the kinetics of autoagglutination. These assays were performed in triplicate with three separate biological replicates.

### Electron microscopy

Electron microscopy of wild type and *csrA* mutant *C*. *jejuni* was performed essentially as described [21]. Briefly, cells were grown on MH agar for 16 hours. After growth, wild-type and *csrA* mutant strains were resuspended from MH agar into PBS, pelleted for 3 min at 13,200 r.p.m. in a microcentrifuge, resuspended in 2% gluteraldehye, and then incubated on ice for 1 h. Samples were then stained with 2% uranyl acetate and visualized with an FEI Technai G2 Spirit Bio TWIN transmission electron microscope.

### Mouse colonization

Competition mouse colonization studies were carried out as previously described [16]. Wild-type and *csrA* mutant were mixed in equal proportions (approximately 5 x 10^8^ CFU each) prior to inoculation and administered to BALB/cByJ mice (The Jackson Laboratory) by oral gavage. Fecal pellets where collected from each infected mouse at 0, 7, 14, and 21 days post-inoculation and suspended in PBS. Shed bacteria were then enumerated via serial dilution of fecal suspensions on MH agar containing 5% sheep blood, cephaperazone (20 μg/ml), vancomycin (10 μg/ml) and amphotericin B (2 μg/ml) without or with chloramphenicol (15 μg/ml) to differentiate between wild-type and mutant bacteria recovered. Mouse colonization experiments were approved by the Augusta University Institutional Animal Care and Use Committee (IACUC)–protocol 09-02-168. Mice do not suffer any symptoms or disease resulting from the colonization, and were observed daily to ensure that no adverse events occurred (e.g. changes in behavior, activity, posture, or ability to move). At the conclusion of the experiments, mice were euthanized by CO_2_ overdose as recommended by the IACUC, in accordance with the recommendations of the American Veterinary Medical Association Panel on Euthanasia.

### Statistical analysis

Results are presented as means ± standard error of means. Statistical analysis was determined using one-way analysis of variance (ANOVA) or student’s *t*-test. *P* values of less than 0.05 were considered significant.

## Results

### Mutation of CsrA alters protein expression in both mid-log and stationary phase

Considering the pleiotropic phenotypes of the *C*. *jejuni csrA* mutant [5], we performed proteomics experiments to examine the protein expression profiles of the wild type, *csrA* mutant, and complemented mutant strains. *C*. *jejuni* cells were grown overnight at 42°C diluted into fresh MH media and then allowed to grow in parallel to either mid-log phase (OD_600_ = 0.5) or to stationary phase (OD_600_ = 1.0) at 42°C. These cultures were then harvested and subjected to 2D-DIGE analysis followed by MALDI-ToF/ToF mass spectrometry to enumerate and identify proteins differentially expressed more than ±1.5 fold in the *csrA* mutant bacteria, as compared to the wild type, at both growth phases. We observed that when grown to mid-log phase, 25 proteins showed altered expression in the *csrA* mutant as compared to the wild type; 9 were more highly expressed (Table 2, Table A in S1 File) and 16 were less abundant (Table 2, Table B in S1 File). In contrast, when grown to stationary phase the expression of 111 proteins was altered—54 proteins were more abundant in the *csrA* mutant as compared to the wild type (Table 3, Table C in S1 File), while 57 proteins were less abundant (Table 3, Table D in S1 File). Expression of all of the proteins whose abundance was altered in the *csrA* mutant was restored to normal in the complemented strain. All differentially expressed proteins were excised and subjected to MALDI-ToF/ToF mass spectrometry for identification. The magnitude of change for the majority of the differentially expressed proteins was less than three-fold, consistent with the degree of change of *E*. *coli* CsrA-regulated proteins [22].

**Table 2:** Proteins with altered expression *ΔcsrA* at mid-log phase.

Functional category^1^	Altered expression in *ΔcsrA* (25)	
	Higher (9)	Lower (16)
**Metabolism**		
Amino acid	HisD	AnsA
Carbohydrate	AcnB	OorA, SucC
Energy		NuoG, Ppa
Nucleotide	GuaB	Adk
**Genetic information processing**		
Translation	Tig	EF-P, EF-Tu
Folding, sorting, degradation	HtrA, PEB4	DnaK, GroEL
**Environmental information processing**		
Membrane transport	CjaA	
Signal transduction		CosR
**Cellular processes**		
Cell motility	FlaA, FlaB	Tlp6
Oxidoreductase		AhpC, TrxB
Hypothetical		Cjj81176_0443, Cjj81176_0107

^1^KEGG category

**Table 3:** Proteins with altered expression *ΔcsrA* at stationary phase.

Functional category^1^	Altered expression in *ΔcsrA* (111)	
	Higher (54)	Lower (57)
**Metabolism**		
Amino acid	AnsA, GGT, PEB1a, AspA, GlnH, MetY	PheA, IlvC, IlvE, Asd, ArgG, DapA
Carbohydrate	AckA, AcnB, Acs, Cjj81176_0110, FbaA, FumC, Mez, OorA, OorB, OorC, PFOR, Pyk, SucC	AccA, Eno, FrdA, GltA, OorD, Pta, SucD
Energy	NuoI	AtpA, AtpD, Cj0414, MfrA, TorA
Nucleotide	GuaB	Adk, PurA, PurB
Glycan		LpxB, LgtF, WaaF
Cofactors/vitamins	PabB	Cjj81176_0265, CoaE
Other secondary metabolites		Cft
**Genetic information processing**		
Transcription	Rho	
Translation	EF-Tu, HisS, RpmB, SerS, Tig	FusA, LepA, RpsA
Folding, sorting, degradation	HtrA, PEB4	FtsH, GroEL, HtpG, MogA
Replication and repair	Ogt, RecA, RuvA	
**Environmental information processing**		
Membrane transport	Cjj81176_1525, Cjj81176_1566, ModC, PorA	Cjj81176_0211, PstB
Signal transduction	CosR, Fur, RacR	
**Cellular processes**		
Cell motility	FlaA, FlaB, FliD, PseI, Tlp6, Tlp8	CheV, CheY
Oxidoreductase	Cjj81176_0382	AhpC, KatA, Tpx, TrxA, TrxB
Adhesins^2^	PEB3	PEB2, CadF, FlpA, Cjj81176_1348
Hypothetical	pVir08, Cjj81176_1382, Cjj81176_1458, Cjj81176_0107, Cjj81176_0176, Cjj81176_1344	Cjj81176_1215, Cjj81176_0435, Cjj81176_0977, Cjj81176_0792, Cjj81176_0793, Cjj81176_0828, Cjj81176_0443, Cjj81176_0729, Cjj81176_0266, Cjj81176_1062

^1^KEGG category;

^2^ Known *C*. *jejuni* adhesins

The proteins whose expression was altered in the *csrA* mutant fell into a number of functional classes, including central metabolism (amino acid metabolism, TCA cycle, acetate metabolism, glycolysis, gluconeogenesis), respiration, transporters, and heat shock proteins (Tables 2 and 3). Other functional categories included those related to pathogenesis, such as motility / chemotaxis, oxidative stress resistance, and cell wall factors including adhesins. At mid-log phase, two of the proteins most overexpressed in the *csrA* mutant were FlaA and FlaB flagellins, along with HtrA protease and the TCA cycle protein AcnB (Table 2). Proteins with lower expression in the *csrA* mutant include the oxidative stress proteins AhpC and TrxB, the response regulator CosR, and the chemoreceptor Tlp6.

A much greater number of proteins showed differential expression in the *csrA* mutant at stationary phase. Among the more highly expressed proteins were several related to amino acid metabolism (AnsA, GGT, PEB1a, AspA, GlnH, and MetY), TCA cycle (AcnB, FumC, OorABC, and SucC), acetate metabolism (AckA and Acs), and several respiration-related proteins. A number of proteins related to heat shock or cell processes were also overexpressed. Three global regulators (CosR, Fur, and RacR) were also more abundant in the *csrA* mutant, as well as several motility / chemotaxis proteins including FlaA and FlaB, which were the most highly overexpressed proteins in the *csrA* mutant (Table 3).

Among proteins whose expression was lower in the *csrA* mutant were several other amino acid metabolism (PheA, IlvC, IlvE, Asd, ArgG, DapA) and TCA cycle proteins (FrdA, GltA, OorD, and SucD), as well as the Pta protein, involved in acetate metabolism. Several respiration-related proteins (TorA, MfrA, and Cj0414) were also less abundant. Expression of proteins involved in chemotaxis (CheV, CheY), oxidative stress (AhpC, Tpx, TrxA, TrxB, and KatA) and cell wall structures including fibronectin-binding adhesins (CadF, FlpA, and Cjj81176_1348) was also lower in the *csrA* mutant.

### FlaA expression is regulated by growth-phase and by CsrA

**Fig 1:**
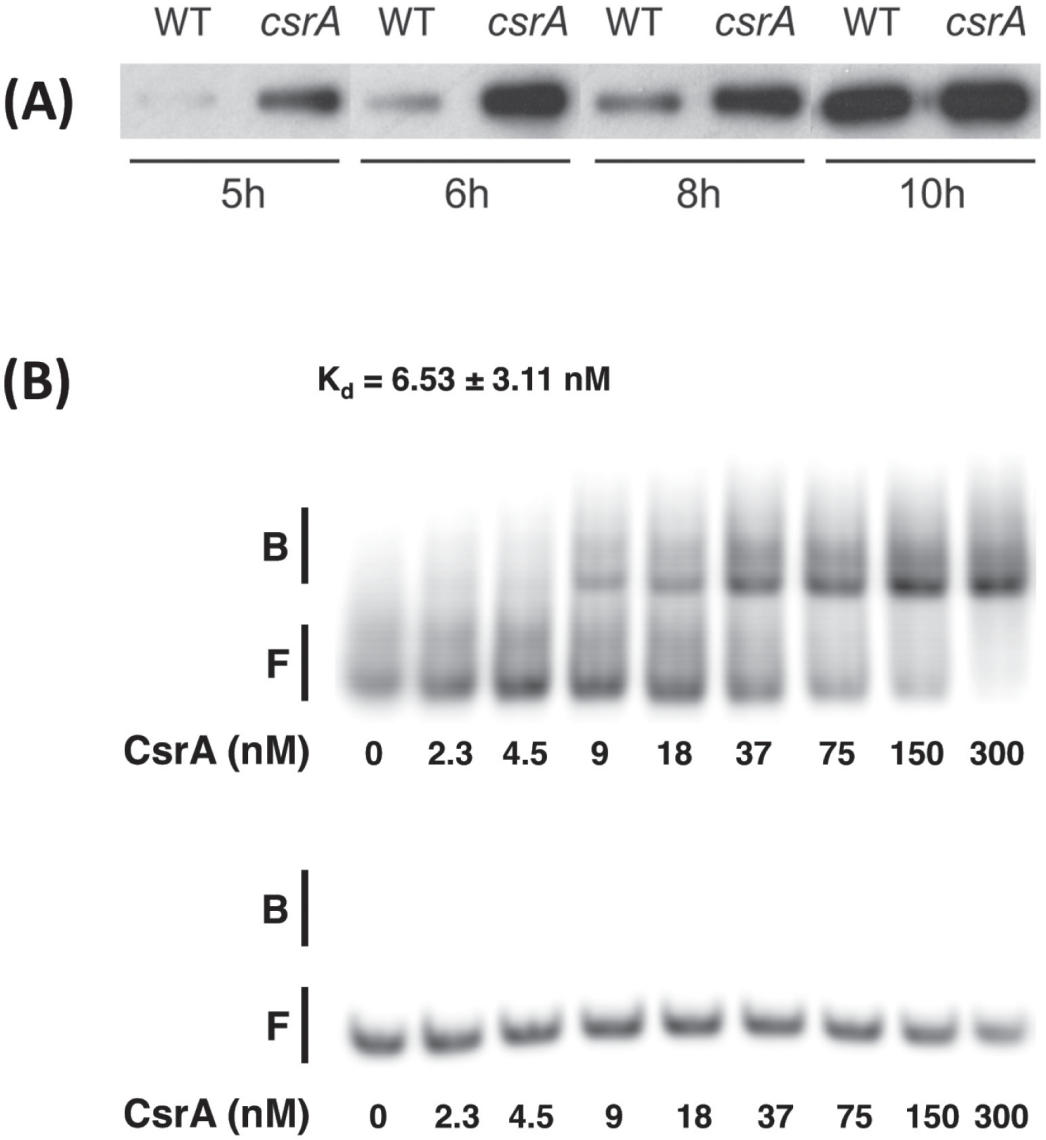
*C*. *jejuni* CsrA regulates FlaA expression by binding to the 5’ end of *flaA* mRNA. **(A)** WT and *csrA* mutant *C*. *jejuni* were diluted to an initial OD_600_ = 0.1 and grown at 42°C. Cells were removed at 5, 6, 8 and 10 hours, and were used in western blots with antibodies against FlaA. **(B)** Electrophoretic Mobility Shift Assay (EMSA) analysis was used to assess the interaction between purified *C*. *jejuni* CsrA-His_6_ and the 5’ end of *flaA* mRNA. Purified CsrA-His_6_ at concentrations varying from 0–300 nM were incubated with 100 ng of purified ^32^P-end-labeled RNA corresponding to the 5’ ends of either *C*. *jejuni flaA* (top panel) or *E*. *coli phoB* (bottom panel) as a control that does not bind CsrA [15]. The positions of bound (B) and free (F) RNA are shown.

Proteomics results showed increased expression of FlaA in the *csrA* mutant, at both mid-log and stationary phase (Tables 2 and 3). To confirm and extend these results, we used western blots to assess FlaA expression at several growth time points. The expression of FlaA in WT cells increased significantly thoughout the growth curve, with much greater FlaA levels at 10 hours of incubation as compared to 5 hours (Fig 1A). Like WT, expression of FlaA in the *csrA* mutant was growth–phase regulated, with highest amounts of FlaA seen in later stages of growth. Consistent with proteomics results, the expression of FlaA was greater in the *csrA* mutant than in the WT strain (Fig 1A). This suggests that CsrA is an inhibitor of FlaA expression.

### CsrA binds directly to *flaA* RNA

Because proteomics and western blots both implicated CsrA in regulation of flagellar expression, we performed EMSAs to determine whether CsrA directly regulated FlaA expression by binding to its mRNA. We generated RNA transcripts corresponding to the 5’ end of *flaA* mRNA, as well as to *E*. *coli phoB* as a negative control that does not bind CsrA [15]. These transcripts were incubated with purified CsrA-His_6_ at concentrations ranging from 0–300 nM. CsrA binding was observed for *flaA* but not *phoB* RNA (Fig 1B). CsrA bound to *flaA* mRNA with a dissociation constant of 6.53 +/- 3.11 nM, indicating a specific, high-affinity interaction.

### Autoagglutination is altered in the absence of CsrA

**Fig 2:**
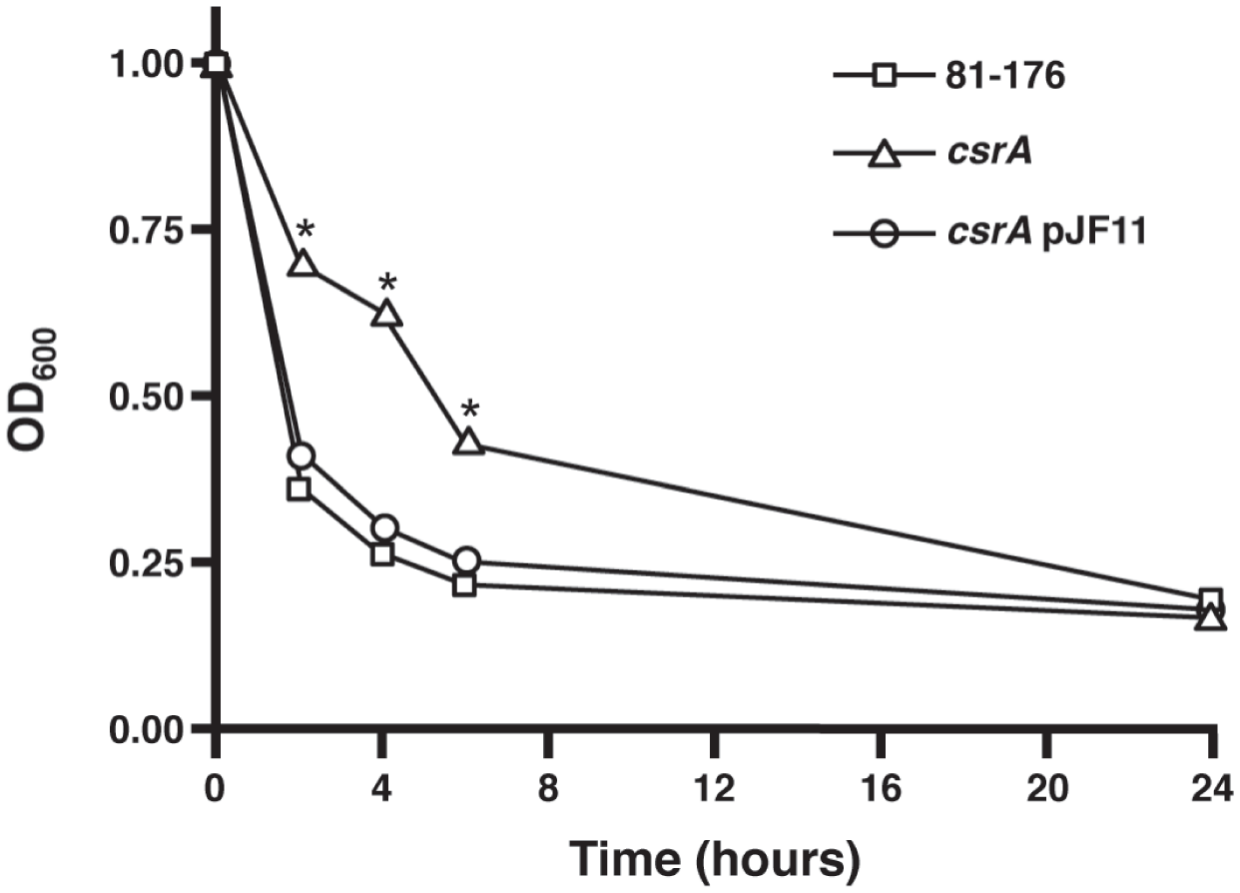
The kinetics of autoagglutination are altered in the *csrA* mutant strain. Static suspensions of *C*. *jejuni* wild type, *csrA* mutant, and complemented *csrA* mutant strains were incubated and OD_600_ measurements were taken at 2, 4, 6, and 24 hours. The assay was performed in triplicate on three separate occasions. Statistical significance (p<0.05) is represented by an asterisk. Error bars are present; however, they are too small to be seen.

**Fig 3:**
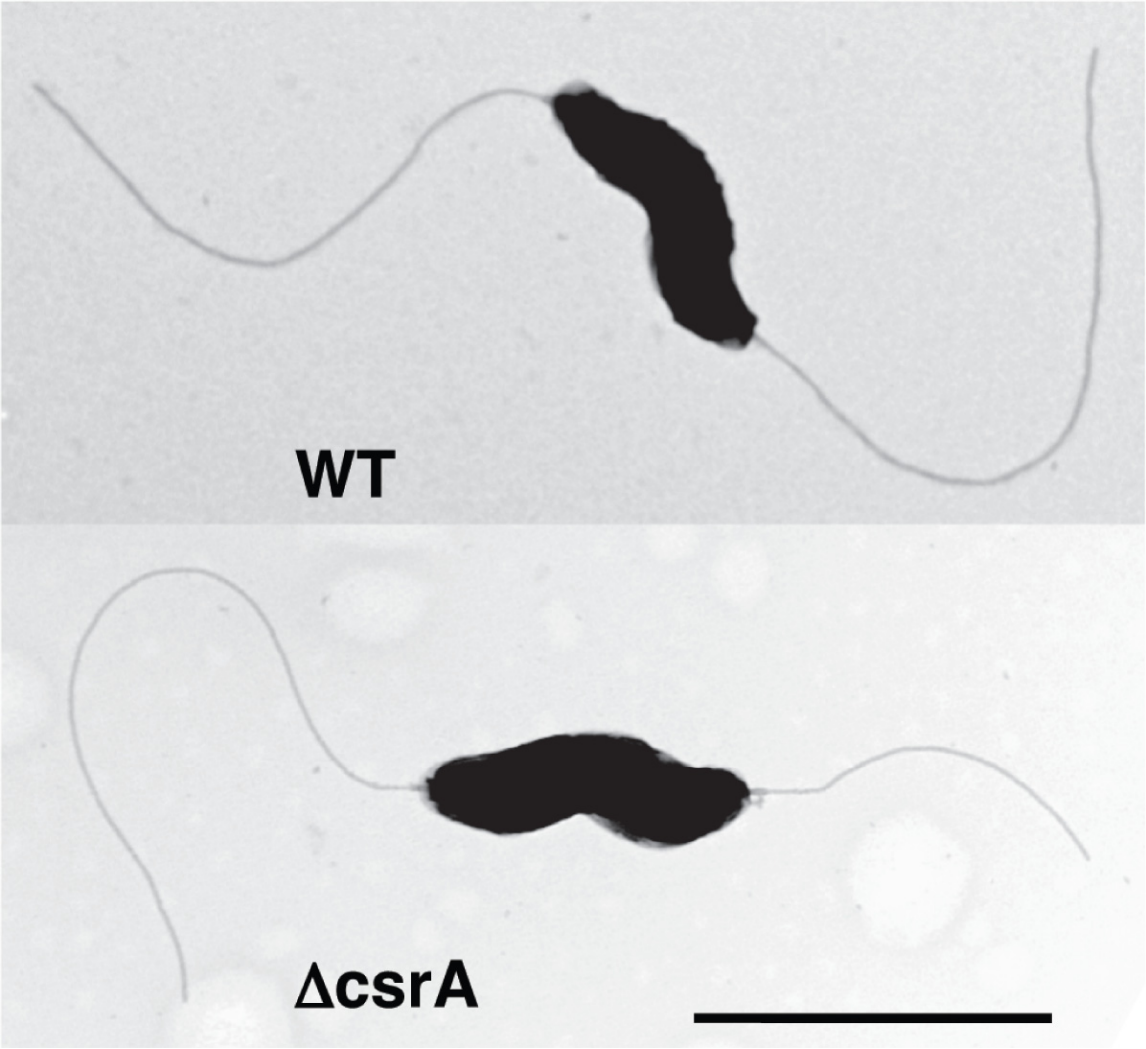
Mutation of *csrA* does not affect flagellar structure. Transmission electron microscopy of wild type (top) and *csrA* mutant (bottom) strains showed that flagellar structure is not affected by the *csrA* mutation. The bar at the lower right of the figure is 1 μm.

Proteomic analysis of the *csrA* mutant compared to the wild type strain revealed altered expression of PEB4. Previous studies in our laboratory revealed that mutation of PEB4 changed the ability of *C*. *jejuni* cells to autoagglutinate, a phenotype dependent on full length, glycosylated flagella [20]. We examined the kinetics of autoagglutination over a 24 hour period and found that the rate of autoagglutination was different between the wild type, *csrA* mutant, and complemented mutant strains (Fig 2). Although all three strains autoagglutinated to similar levels after 24 hours, at 2, 4 and 6 hours, the *csrA* mutant autoagglutinated to a lesser extent compared to the wild type and complemented strains (p<0.05), indicating that mutation of *csrA* resulted in delayed autoagglutination. Electron microscopy of WT and *csrA* mutant strains showed no overt differences in flagellar number, structure, or length (data not shown) that would explain differences in autoagglutination or motility [5](Fig 3).

### Mutation of *csrA* renders *C*. *jejuni* defective in its ability to colonize mice

**Fig 4:**
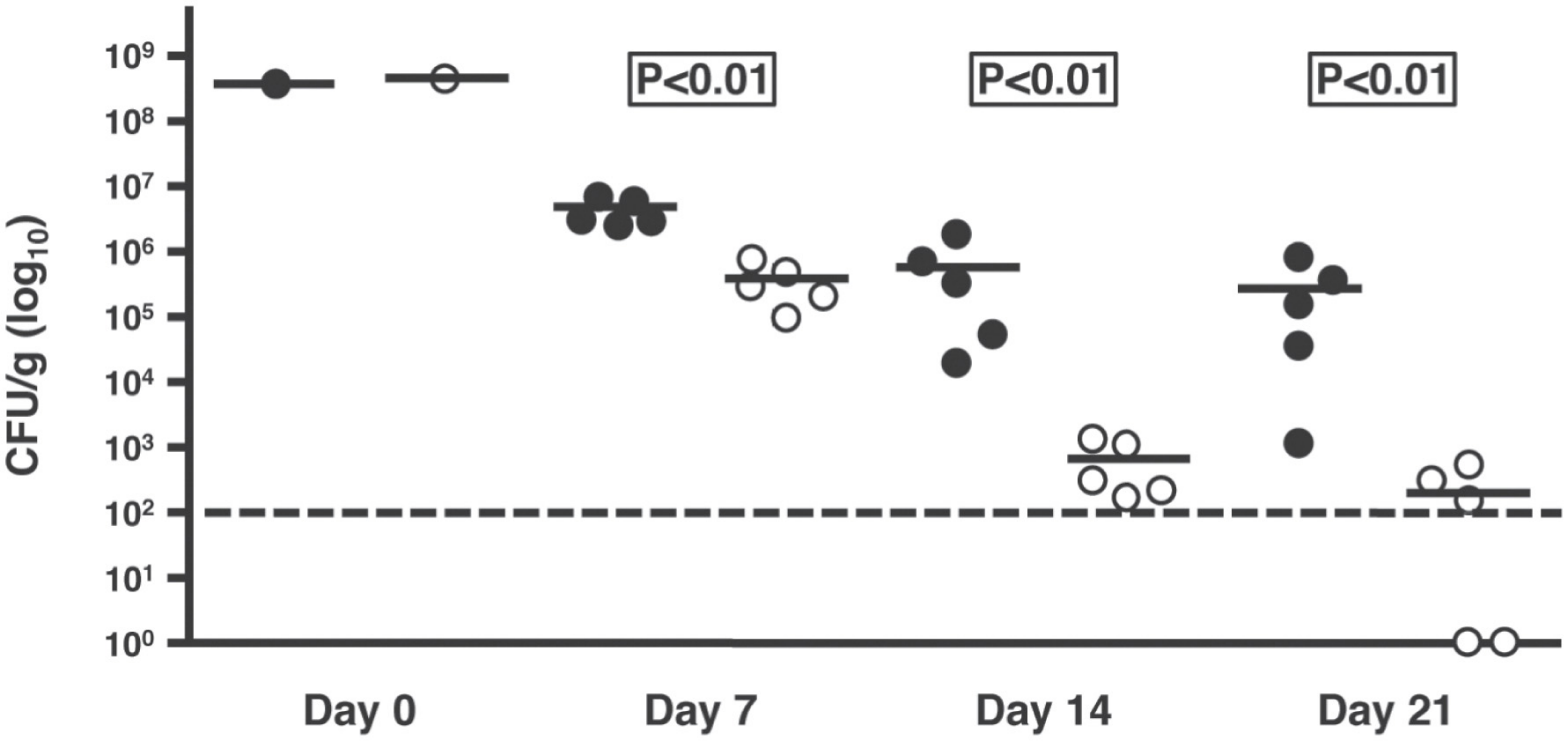
CsrA is involved in mouse colonization. BALB/c-ByJ mice were orally inoculated with a 1:1 mixture of wild type (closed circles) and *csrA* mutant (open circles) *C*. *jejuni* and fecal pellets were collected at 7, 14, and 21 days post-infection for enumeration of wild type and mutant bacteria. The geometric means of colonization of each group are represented by horizontal lines. The limit of detection (represented by a dashed line) was 10^2^ CFU/g.

Numerous lines of evidence suggest defective pathogenicity of the *csrA* mutant [5]. Therefore, we tested the ability of the *csrA* mutant to colonize mice using a competitive infection model as previously described [16]. Wild type and *csrA* mutant bacteria were mixed at a 1:1 ratio and orogastrically administered to BALB/c-ByJ mice. Fecal pellets were collected at 7, 14, and 21 days post-inoculation, the pellets were homogenized in PBS and bacteria shed were serially diluted and enumerated on selective and non selective MH agar (Fig 4). The mean colonization densities of both strains were compared and a significant decrease in the ability of the *csrA* mutant strain to compete against the wild type was observed. This deficiency was observed at 7, 14, and 21 days post-infection (p<0.01 for each) and became more striking as the experiment progressed. The parent cultures from both strains used for mouse infection were tested for motility on the day of infection to rule out phase variation in flagellar synthesis as a factor in mouse colonization (data not shown) and both strains have been shown to grow at similar rates [5], indicating that the colonization defect of the *csrA* mutant also was not due to a general growth defect.

## Discussion

Paradoxically, *C*. *jejuni* survives in diverse environments despite its relative small number of regulatory proteins. While several transcriptional regulators have been characterized, our understanding of how gene regulation affects *C*. *jejuni* disease is still incomplete, prompting us to investigate the post-transcriptional regulator CsrA and its role in pathogenesis. Previously, we reported that mutation of *csrA* in the highly virulent *C*. *jejuni* strain 81–176 altered motility, biofilm formation, oxidative stress responses, and *in vitro* adherence and invasion of intestinal epithelial cells [5]. In the current study, we examined the proteomes of WT, isogenic *csrA* mutant and complemented *csrA* mutant strains, and identified a role for CsrA in the expression of a number of proteins important to *C*. *jejuni* central metabolism, motility, oxidative stress resistance, and pathogenesis.

A total of 117 unique proteins were differentially expressed in the *csrA* mutant relative to wild type strain 81–176, and these comprise the putative CsrA regulon. A greater number of proteins showed altered abundance at stationary phase (111 proteins) compared to mid-log phase (25 proteins); a total of 19 proteins were differentially expressed in the *csrA* mutant at both stages of growth. Expression of each of these proteins was restored to wild type levels in the complemented mutant. Together, these data suggest that CsrA regulation is more predominant during stationary phase, consistent with the role of CsrA as a stationary phase regulator in other bacteria [23]. The degree of regulation (generally 1.5- to 4-fold) noted for proteins of the presumptive CsrA regulon is similar to those of proteins regulated by *E*. *coli* CsrA [22]. This is consistent with the role of CsrA in fine-tuning protein expression; this level of regulation can be superimposed on transcriptional and/or allosteric regulation. In this work we examined differential protein expression in the *csrA* mutant at 42°C, the relevant growth temperature for the thermophilic *C*. *jejuni*, however, it is certainly possible that varying the growth temperature or other environmental conditions could have an effect on CsrA regulation.

Proteins of the putative CsrA regulon fall into a number of different functional classes. Consistent with proteins regulated by CsrA in *E*. *coli* [22, 24] and other bacteria, by far the largest class of proteins putatively regulated by *C*. *jejuni* CsrA is related to central metabolism. Among *C*. *jejuni* CsrA targets, many were involved in carbon metabolism. While the effect of CsrA on the expression of amino acid metabolizing proteins is modest at mid-log phase (two proteins), at stationary phase there are 12 such proteins whose expression is altered. Proteins with higher expression (i.e. normally repressed by CsrA) are members of pathways for acquiring and metabolizing aspartate, glutamate, asparagine, and glutamine, which are some of the preferred carbon sources for *C*. *jejuni* [25, 26]. Proteins involved in metabolism of aromatic and branched-chain amino acids, lysine, and arginine are expressed at lower amounts in the *csrA* mutant, suggesting that in wild-type cells CsrA stimulates their expression.

*C*. *jejuni* lacks a complete glycolytic pathway due to the absence of 6-phophofructokinase [25, 26]. However, the FbaA protein, which functions in both glycolysis and gluconeogenesis, is more highly expressed in the *csrA* mutant. In the *csrA* mutant, several proteins involved in the flow of carbon through the lower end of glycolysis and into the TCA cycle are altered in stationary phase–Pyk (pyruvate kinase)[27] and Mez (malic enzyme)[27] are more highly expressed, while Eno (enolase) is less abundant. Expression of 10 proteins of the TCA cycle was altered in the *csrA* mutant. AcnB, FumC, OorA, OorB, OorC, and SucC levels were elevated at stationary phase, while those of FrdA, GltA, OorD, and SucD were lower. The expression of OorA is lower in the *csrA* mutant during mid-log phase, suggesting that control of TCA cycle enzymes by CsrA is complex. Abundance of a number of proteins that are involved in providing electrons for respiration is also altered in the *csrA* mutant, some with greater and some with lesser expression. NuoI [28] and PFOR [29] are more highly expressed during stationary phase, while the respiratory proteins MfrA [30–32], TorA [32, 33], and Cj0414 [16] are less abundant. The altered expression of these proteins suggests that CsrA controls aspects of cellular respiration, especially at stationary phase.

Three putative CsrA targets (AckA, Acs, and Pta) are related to acetate metabolism (see also below). Like *E*. *coli*, *C*. *jejuni* has an acetate switch [34], whereby excess carbon accumulated during log-phase growth is converted to acetate via the Pta and AckA proteins. Upon consumption of preferred carbon sources, at the transition into stationary phase *C*. *jejuni* switches from acetate production to consumption via either AckA-Pta or Acs. In the *csrA* mutant, AckA and Acs are more highly expressed at stationary phase while Pta abundance is lower. Because this pathway is reversible, there exists the possibility that CsrA could regulate production and/or consumption of acetate. Together, these results indicate that CsrA controls a number of aspects of central metabolism, primarily during stationary phase, and may allow the fine-tuning of nutrient assimilation, carbon flux, and respiration.

CsrA also appears to regulate a number of other cellular processes (Tables 2 and 3 and Tables A-D in S1 File), including predicted transporters, biosynthesis of cofactors and vitamins, transcription and translation, nucleotide metabolism, DNA repair, and energy homeostasis. Heat shock proteins or chaperones such as GroEL, HtpG, HtrA, and PEB4 are also among the CsrA regulon, suggesting CsrA regulation of general stress responses at both mid-log and stationary phases. Some of these have also been shown to play a role in virulence. PEB4 is a periplasmic peptidyl cis-trans isomerase, and mutation of the *peb4* gene results in *C*. *jejuni* that are more motile and more invasive of INT407 human intestinal epithelial cells, and are less able to colonize mice than 81–176 [35]. *C*. *jejuni* HtrA is associated with aerotolerance, adherence to and invasion of INT407 cells [36], altered host cell apoptosis and intestinal immune responses [37] and cleaves E-cadherin [38]. It is possible that alteration of one or more of these proteins impact the phenotypes previously shown for the *csrA* mutant [5]. Clearly a full understanding of the influence of CsrA in central metabolism and other cellular processes will be a substantial undertaking and the subject of future research.

Three proteins (Fur, CosR, and RacR) whose expression is increased in the *csrA* mutant are transcriptional regulators. These proteins direct large regulatory networks related to *C*. *jejuni* iron availabilty, oxidative stress resistance, and growth temperature, respectively. Fur regulates both Fe homeostasis and oxidative stress resistance responses; the Fur regulon includes KatA, AhpC, and TrxB [39] whose expression is also altered in the *csrA* mutant. However, other than these proteins, there is not significant overlap of the Fur regulon with that of CsrA, suggesting that CsrA regulation of KatA, AhpC, and TrxB is not mediated simply through Fur. CosR is an essential orphan response regulator that is responsible for regulating the expression of 93 genes including several that are also differentially expressed in the *csrA* mutant such as KatA, AhpC, TrxB, SucCD, and GroEL [40]. AhpC transcription is not significantly affected by CosR knockdown, leading Hwang et al. to conclude that AhpC is likely also regulated post-transcriptionally [40]; our present data suggests that this may be mediated by CsrA. RacR is a transcriptional regulator that controls the expression of a number of genes in response to growth temperature [41–43]. Although some of the RacR targets are also members of the CsrA regulon, including catalase, cytochrome c peroxidase, AnsA, AspA, Tlp6, and GGT [41–43], the majority are not. As with Fur, the lack of overlap among the majority of proteins in the CosR, RacR and CsrA regulons shows that CsrA effects are not manifest solely through CosR or RacR. Therefore, while altered expression of these regulators in the *csrA* mutant raises the possibility that some of the effects of CsrA on whole-cell protein expression are indirect (i.e. mediated through secondary regulators), the lack of significant overlap among members of these regulons suggest that most CsrA-related changes in protein expression are direct. This is consistent with results found with *E*. *coli* CsrA [22]. However, it is possible that the expression of some of the targets of these transcriptional regulators is modulated by CsrA.

Another class of targets putatively regulated by CsrA and directly relevant to pathogenesis is motility and chemotaxis. FlaA and FlaB are the major and minor flagellins, respectively, that make up *C*. *jejuni* flagella [44]. The expression of both FlaA and FlaB is significantly higher at both mid-log and stationary phase (3.4- to 5.4-fold), and this magnitude of regulation is among the highest noted for any CsrA target in these experiments. Greater expression of FlaA in the *csrA* mutant at various stages of growth was confirmed using western blots (Fig 1A). We also observed growth-phase regulation of FlaA expression, with FlaA abundance increasing significantly throughout the growth curve (Fig 1A) in both WT and *csrA* mutant strains. Using EMSA (Fig 1B), we showed that CsrA directly binds to the 5’ end of *flaA* mRNA containing the *flaA* RBS; binding of CsrA in this region is consistent with the model of CsrA function in other bacteria [23]. Together, these data show that CsrA directly represses FlaA synthesis by binding to the 5’ end of *flaA* mRNA, and that FlaA abundance is growth-phase-dependent. The greater expression of FlaA and FlaB seems paradoxical with respect to the decreased motility of the *csrA* mutant [5]. Electron microscopy of the *csrA* mutant shows that the *csrA* mutant has flagella that appear normal in number, length, and morphology (Fig 3), suggesting that there are no obvious flagellar defects that would explain the lesser motility of the *csrA* mutant. It is possible that there are subtle differences in the flagella of the *csrA* mutant that are not apparent by using TEM that could result in the altered autoagglutination kinetics of this strain (Fig 2), which depend on glycosylated flagella [45]. The apparent regulation of *C*. *jejuni* flagellins is consistent with CsrA being an ancestral regulator linking motility and cellular metabolic processes, as proposed for *B*. *subtilis* [46] and with the proteomics results described in this work.

The expression of several chemotaxis-related proteins is also altered in the *csrA* mutant, including two methyl-accepting chemotaxis proteins, Tlp6 and Tlp8. The abundance of Tlp6 is lower at mid-log phase, but the expression of both Tlp6 and Tlp8 is higher at stationary phase. Both Tlp6 and Tlp8 are group C sensors [47], which are thought to recognize cytoplasmic signals, such as redox status in the case of Tlp8 [48]. Furthermore, the chemotaxis signaling proteins CheV and CheY are both expressed at lower levels in the *csrA* mutant, but only at stationary phase. CheY is a response regulator that controls the direction of flagellar rotation in response to chemotactic signals [44]. CheV is another component of the *C*. *jejuni* chemotaxis apparatus, mediating adaptation to chemoattractants [44]. CheV has affinity for both Tlp6 and Tlp8 [49], suggesting that interplay between these proteins and downstream chemotaxis signaling events could be influenced by CsrA. Due to changes in the expression of multiple chemotaxis proteins, it is therefore possible that chemotaxis is altered in the *csrA* mutant, which could contribute to the altered motility of the *csrA* mutant. Alternatively, the altered expression of characteristics not typically associated with motility could play a role, including oxidative stress resistance.

Several proteins whose expression is altered in the *csrA* mutant are related to oxidative stress, and could explain the increased oxidative stress sensitivity of the mutant strain [5]. A number of antioxidant pathways are known to be important for oxidative stress responses, particularly to aerotolerance [50–56], and here we show that the abundance of some of these is altered in the absence of CsrA. The expression of KatA, Tpx, AhpC, TrxA, and TrxB is lower in the *csrA* mutant at stationary phase, while AhpC and TrxB are less abundant at mid-log phase as well. KatA [57], and Tpx [55] are the primary defense mechanisms against hydrogen peroxide [57], to which the *C*. *jejuni csrA* mutant shows increased sensitivity [5]. AhpC is involved in defense against organic peroxides [50]. We did not identify the important oxidative stress resistance protein superoxide dismutase as being CsrA-regulated in these experiments. Together, the lowered expression of these antioxidant proteins could explain the sensitivity of the *csrA* mutant to oxidative stress. However, Flint et al. identified a number of other pathways that may be involved indirectly in oxidative stress resistance, including motility, electron transport, energy metabolism, cation transport, and general bacterial physiology [58]. It is conceivable that the decreased motility noted for the *csrA* mutant [5], or changes in respiratory proteins, is associated with the increased sensitivity of the mutant to various oxidative stressors.

Another defect in the *csrA* mutant is a decreased ability to bind to intestinal epithelial cells *in vitro* [5]. *Campylobacter* adherence has been extensively characterized, and a number of adhesins including the fibronectin-binding proteins CadF, FlpA and Cjj81176_1348 have been identified [59, 60]. In the absence of CsrA, all of these adhesins have reduced expression when compared to the wild type, suggesting that these may influence the decreased adherence phenotype observed in the *csrA* mutant. PEB3 is another adhesin [61] that is altered in the *csrA* mutant, however, its expression is increased at stationary phase and is therefore unlikely to be the cause of the adherence defect of the *csrA* mutant [5].

The *csrA* mutant also exhibits a defect in biofilm production [5], and one of our goals is to determine the role of CsrA in biofilm formation. The effects of CsrA on biofilm accumulation in *E*. *coli* and other bacteria have been well documented [62–66]. While the study of biofilms in *C*. *jejuni* is still emerging and the precise role of CsrA remains unknown, a number of factors and proteins are known to contribute to *C*. *jejuni* biofilm formation. While the biofilm-related *E*. *coli pgaABCD* operon [66] is absent from *C*. *jejuni*, recently an α-dextran was reported to play a role in forming *C*. *jejuni* biofilm [13], although the genes responsible for the synthesis of this polysaccharide have not been defined. Other known glycans are not required for *C*. *jejuni* biofilm accumulation, as mutants lacking capsule, protein glycosylation, or full-length lipooligosaccharide formed biofilms at levels equal to or greater than wild type [45, 67].

A previous proteome study from Kalmokoff et al. examined *C*. *jejuni* NCTC 11168 proteins that were upregulated in biofilms compared to planktonic bacteria and identified a major role for motility-related proteins in biofilm production [68]; the role of motility in biofilm formation was confirmed and extended in subsequent work [12]. Of the biofilm-related proteins identified by Kalmokoff [68], 13 are also in the CsrA regulon identified in the current work: FlaA, FlaB, FliD, AhpC, Tpx, GroEL, CosR, RacR, SucD, PEB1a, PEB4, Cjj81176_0443, and Cjj81176_0793. There are several possibilities that could explain the overlap in the subset of proteins with altered expression in biofilms and those regulated by *csrA*. The most simple explanation is that CsrA plays a role in regulating protein expression in biofilms, as in other bacteria CsrA is a central regulator coordinating the transition from planktonic (motile) to biofilm (sessile) growth [23, 63]. In *C*. *jejuni*, the regulation of major flagellin FlaA (as well as minor flagellin FlaB), is also consistent with previous observations on the role of flagella and motility in biofilm formation [12, 68]. Combined with the observation that *C*. *jejuni* CsrA protein regulation is more active at stationary phase than at mid-log, this suggests that CsrA may play a direct role in biofilm development upon entering stationary phase.

As discussed previously, during the *C*. *jejuni* acetate switch, excess carbon produced during logarithmic growth is converted to acetate, which is then used as an alternate carbon source upon consumption of preferred carbon sources [34]. Because the timing of the onset of *C*. *jejuni* biofilm production coincides with the period of acetate consumption [34], it is possible that CsrA-mediated modulation of the expression of acetate metabolizing enzymes such as AckA, Pta, and Acs could influence biofilm formation. As acetate metabolism is involved in the production of *E*. *coli* biofilms [69, 70], the altered expression of *C*. *jejuni* proteins related to acetate metabolism is intriguing. Acetate metabolism could play a role *C*. *jejuni* biofilms either as a stationary phase carbon source, required for synthesizing a biofilm polysaccharide [13] via gluconeogenesis (including CsrA-regulated FbaA) or via the acetate pathway intermediate acetyl phosphate (AcP). In *E*. *coli* and other bacteria, AcP can phosphorylate response regulators, resulting in changes of the downstream targets of these response regulators, including genes involved in biofilm formation [71, 72]. Interestingly, AckA is elevated considerably in the *csrA* mutant at stationary phase, while Pta expression is less abundant. This suggests differential regulation of these two enzymes, and thereby a possible mechanism for modulation of AcP levels. In *C*. *jejuni*, the response regulator CprS [7] has been implicated in biofilm formation and therefore represents one such target, although its expression was not detected as altered in the *csrA* mutant. Inspection of the 25 unique proteins that are regulated by CprS reveals some overlap with those regulated by CsrA; there are a total of 16 proteins that are regulated by both CsrA and CprS (FumC, Fba, Asd, SucD, HisS, RpsA, EF-P, EF-Tu, Tig, MOMP, FlaA, AhpC, KatA, TrxB, Cjj81176_0729 (Cj0706), and CosR)[7]. It is therefore possible that the effect of CsrA on biofilm formation is mediated in part through modulation of CprS activity, perhaps by AcP-mediated phosphorylation.

A large number of transcriptional changes occur as *C*. *jejuni* transitions into stationary phase, concordant with the acetate switch [34], although *csrA* expression was not altered. It is therefore possible that *csrA* is not transcriptionally regulated in *C*. *jejuni* and its activity is instead modulated in stationary phase by interaction with other regulatory molecules, although evidence suggests that sRNAs (e.g. *csrB*, *csrC*) typically found in CsrA regulatory systems are not involved in controlling CsrA activity in *C*. *jejuni* [73]. It was also observed that as *C*. *jejuni* transitions into stationary phase, there is a burst of motility [34]. Our previous study indicated that CsrA is an activator of motility in *C*. *jejuni* [5]. As cells enter into stationary phase extracellular signals such as acetate concentration may trigger CsrA regulation of motility and chemotaxis in order for *C*. *jejuni* to search for new carbon sources. The potential role of CsrA as a regulator of stationary phase traits may suggest that it compensates for the lack in *C*. *jejuni* of RpoS, the enteric stationary phase sigma factor, and by data suggesting that similar classes of genes differentially expressed in the *csrA* mutant are also altered in the switch from logarithmic growth to stationary phase. We acknowledge that the current work identifies CsrA-related expression differences only in strain 81–176, and it will be interesting in future work to examine whether there are strain-specific changes in CsrA regulation.

Finally, considering the number of metabolic and virulence-associated phenotypes affected by mutation of *csrA* and the proteins differentially expressed in the mutant, we administered a 1:1 mixture of *csrA* mutant and wild type bacteria to mice to determine whether CsrA was involved in mouse colonization. While CsrA was not absolutely essential, we showed that CsrA is required for full colonization of this mammalian host. When the mutant competed poorly with the wild type and did not colonize the mice to the same extent as wild type. Given the pleiotropic nature of the *csrA* mutant strain, it is difficult to assign this colonization deficiency to one specific pathway or phenotype. It is possible that the competitive colonization defect of the *csrA* mutant reflects an overall fitness of the mutant, although its *in vitro* growth is similar to that of wild type. However, *csrA* is upregulated in a rabbit ileal loop model, suggesting that the expression of CsrA is involved in *C*. *jejuni* colonization of the mammalian intestine [74]. We also note that the body temperature of the mouse (37°C) is different than the temperature at which the proteomics experiments were done (42°C), so we cannot preclude that growth temperature of the thermophilic *C*. *jejuni* has an effect on the colonization results.

In summary, our data indicate that CsrA participates in a complex remodeling of *C*. *jejuni* central metabolism and general stress responses during stationary phase. In addition to metabolic proteins, CsrA affects the expression of a number of proteins with roles in pathogenesis-related functions, such as in motility/chemotaxis, oxidative stress resistance, and host cell interactions. In particular, CsrA is a direct regulator of FlaA expression in a growth-phase dependent manner, and affects autoagglutination without causing a gross defect in flagellar structure. These data suggest that *C*. *jejuni* CsrA plays a role in stationary phase physiology, potentially by fine-tuning the metabolic changes involved in the transition to stationary phase and to biofilm formation.

## Supporting Information

S1 File Proteins with altered expression in the *csrA* mutant. Table A. Proteins with increased expression in the *csrA* mutant—mid-log. Table B. Proteins with decreased expression in the *csrA* mutant—mid-log. Table C. Proteins with increased expression in the *csrA* mutant—stationary. Table D. Proteins with decreased expression in the *csrA* mutant—stationary.
(PDF)
