# Supplementary material for: Campylobacter jejuni CsrA Regulates Metabolic and Virulence Associated Proteins and Is Necessary for Mouse Colonization
Source: PLoS One. 2016 Jun 3;11(6):e0156932. doi: 10.1371/journal.pone.0156932 (PMC4892619; doi:10.1371/journal.pone.0156932)
Supplement: S1 File — Table A. Proteins with increased expression in the csrA mutant—mid-log. Table B. Proteins with decreased expression in the csrA mutant—mid-log. Table C. Proteins with increased expression in the csrA mutant—stationary. Table D. Proteins with decreased expression in the csrA mutant—stationary. (PDF) [file pone.0156932.s001.pdf]

Supporting Information - S1 File

Table A. Proteins with increased expression in the *csrA* mutant – mid-log

Protein	Fold change (ΔcsrA vs. WT)	Functional category
HtrA - serine protease	+4.2	Folding, sorting, degradation
FlaB - minor flagellin	+3.5	Cell motility
FlaA - major flagellin	+3.4	Cell motility
AcnB - aconitate hydratase	+3.0	Carbohydrate metabolism
PEB4 - periplasmic PPIase	+2.1	Folding, sorting, degradation
GuaB - IMP dehydrogenase	+2.0	Nucleotide metabolism
Tig - trigger factor	+2.0	Translation
CjaA – glutamine binding protein	+1.7	Membrane transport
HisD – histidinol dehydrogenase	+1.5	Amino acid metabolism

Total - 9

Table B. Proteins with decreased expression in the *csrA* mutant – mid-log

Protein	Fold change (ΔcsrA vs. WT)	Functional category
Cjj81176_0443 - hypothetical	-3.3	Hypothetical
AhpC - alkyl hydroperoxide reductase	-3.2	Oxidoreductase
GroEL - chaperone	-3.2	Folding, sorting, degradation
EF-Tu – elongation factor Tu	-2.5	Translation
NuoG - NADH dehydrogenase I chain G	-2.4	Energy metabolism
SucC – succinyl coA-synthetase β chain	-2.4	Carbohydrate metabolism
DnaK – heat shock protein	-2.1	Folding, sorting, degradation
AnsA – L-asparaginase	-2.0	Amino acid metabolism
Adk – adenylate kinase	-1.9	Nucleotide metabolism
TrxB – thioredoxin reductase	-1.9	Oxidoreductase
EF-P – elongation factor P	-1.8	Translation
Ppa - inorganic pyrophosphatase	-1.8	Energy metabolism
OorA - 2-oxoglutarate-acceptor oxidoreductase	-1.7	Carbohydrate metabolism
Cjj81176_0107 - hypothetical	-1.6	Hypothetical
CosR – response regulator	-1.6	Signal transduction
Tlp6 - chemoreceptor	-1.6	Cell motility

Total - 16

Table C. Proteins with increased expression in the *csrA* mutant – stationary

Protein	Fold change (ΔcsrA vs. WT)	Functional category
FlaA – major flagellin	+5.4	Cell motility
FlaB – minor flagellin	+5.4	Cell motility
pVir08 - hypothetical	+5.4	Hypothetical
AnsA – L-asparginase	+3.1	Amino acid metabolism
HtrA – serine protease	+3.0	Folding, sorting, degradation
PEB3 – major antigenic peptide	+2.8	Adhesin
Cjj81176_1525 - tungstate ABC transporter	+2.7	Membrane transport
PEB4 – periplasmic PPIase	+2.6	Folding, sorting, degradation
CosR – response regulator	+2.5	Signal transduction
AckA – acetate kinase	+2.4	Carbohydrate metabolism
GGT –γ-glutamyl transpeptidase	+2.4	Amino acid metabolism
Ogt - DNA-protein-cysteine methyltransferase	+2.4	Replication and repair
FbaA - fructose-bisphosphate aldolase	+2.1	Carbohydrate metabolism
PseI – pseudaminic acid synthetase	+2.1	Cell motility
Tig – trigger factor	+2.1	Translation
Fur – ferric uptake regulator	+2.0	Signal transduction
PEB1a – aspartate /glutamate binding protein	+2.0	Amino acid metabolism
AspA – aspartate ammonia lyase	+1.9	Amino acid metabolism
OorA - 2-oxoglutarate-acceptor oxidoreductase	+1.9	Carbohydrate metabolism
Rho – transcription termination factor	+1.9	Transcription
Cjj81176_0382- cytochrome c551 peroxidase	+1.8	Oxidoreductase
Cjj81176_1382 - hypothetical	+1.8	Hypothetical
Cjj81176_1458 - hypothetical	+1.8	Hypothetical
Cjj81176_1566 – peptide ABC transporter	+1.8	Membrane transport
ModC – molybdenum transport protein	+1.8	Membrane transport
NuoI - NADH dehydrogenase I chain I	+1.8	Energy metabolism
OorB - 2-oxoglutarate-acceptor oxidoreductase	+1.8	Carbohydrate metabolism
OorC - 2-oxoglutarate-acceptor oxidoreductase	+1.8	Carbohydrate metabolism
Pyk – pyruvate kinase	+1.8	Carbohydrate metabolism
EF-Tu – elongation factor Tu	+1.7	Translation
GuaB - IMP dehydrogenase	+1.7	Nucleotide metabolism
HisS – histidyl tRNA synthetase	+1.7	Translation
RacR – response regulator	+1.7	Signal transduction
RecA - recombinase	+1.7	Replication and repair
RpmB – 50S ribosomal protein L28	+1.7	Translation
RuvA – DNA helicase	+1.7	Replication and repair
SucC - succinyl coA-synthetase β chain	+1.7	Carbohydrate metabolism
AcnB - aconitate hydratase	+1.6	Carbohydrate metabolism
Cjj81176_0107 - hypothetical	+1.6	Hypothetical
Cjj81176_0110 - NAD-independent lactate dehydrogenase	+1.6	Carbohydrate metabolism
Cjj81176_0176 - hypothetical	+1.6	Hypothetical
FliD – flagellar capping protein	+1.6	Cell motility
GlnH - glutamine binding periplasmic protein	+1.6	Amino acid metabolism
PabB - para-aminobenzoate synthase component I	+1.6	Cofactors/vitamins
PFOR - pyruvate-flavodoxin oxidoreductase	+1.6	Carbohydrate metabolism
PorA – major outer membrane protein	+1.6	Membrane transport
SerS - seryl-tRNA synthetase	+1.6	Translation
Tlp6 - chemoreceptor	+1.6	Cell motility
Tlp8 - chemoreceptor	+1.6	Cell motility
Acs – acetyl-coA-synthetase	+1.5	Carbohydrate metabolism
Cjj81176_1344 - hypothetical	+1.5	Hypothetical
FumC – fumarate hydratase	+1.5	Carbohydrate metabolism

MetY - o-acetylhomoserine (thiol) lyase	+1.5	Amino acid metabolism
Mez – malate oxidoreductase	+1.5	Carbohydrate metabolism

Total - 54

Table D. Proteins with decreased expression in the *csrA* mutant – stationary

Protein	Fold change (ΔcsrA vs. WT)	Functional category
Cjj81176_1215 - lipoprotein, NLPA family	-3.8	Hypothetical
OorD - 2-oxoglutarate-acceptor oxidoreductase	-3.2	Carbohydrate metabolism
PheA - chorismate mutase/prephenate dehydratase	-3.2	Amino acid metabolism
Cjj81176_0211 – iron ABC transporter	-2.9	Membrane transport
TorA - trimethylamine N-oxide reductase	-2.9	Energy metabolism
Eno - enolase	-2.8	Carbohydrate metabolism
AtpA – ATP synthase F1 complex α subunit	-2.6	Energy metabolism
AtpD - ATP synthase F1 complex β subunit	-2.4	Energy metabolism
Cft - nonheme iron-containing ferritin	-2.4	Other secondary metabolites
PEB2	-2.4	Adhesin
Cjj81176_0435 – GTP-binding protein	-2.3	Hypothetical
MfrA - methylmenaquinol:fumarate reductase	-2.3	Energy metabolism
Cjj81176_0977 – DnaJ domain protein	-2.3	Hypothetical
AhpC - alkyl hydroperoxide reductase	-2.2	Oxidoreductase
Tpx – thiol peroxidase	-2.2	Oxidoreductase
Cjj81176_0792 - lipoprotein, NLPA family	-2.1	Hypothetical
FrdA - fumarate reductase flavoprotein subunit	-2.1	Carbohydrate metabolism
TrxA - thioredoxin	-2.1	Oxidoreductase
Cjj81176_0793 - lipoprotein, NLPA family	-2.0	Hypothetical
RpsA – 30S ribosomal protein S1	-2.0	Translation
Cjj81176_0828 - oxidoreductase	-1.9	Hypothetical
FusA – Elongation factor G	-1.9	Translation
GroEL – chaperone	-1.9	Folding, sorting, degradation
TrxB – thioredoxin reductase	-1.9	Oxidoreductase
CadF – fibronectin binding protein	-1.8	Adhesin
Cj0414 – gluconate dehydrogenase	-1.8	Energy metabolism
HtpG - chaperone	-1.8	Folding, sorting, degradation
IlvC - ketol-acid reductoisomerase	-1.8	Amino acid metabolism
KatA - catalase	-1.8	Oxidoreductase
LpxB – lipid A disaccharide synthase	-1.8	Glycan metabolism
Cjj81176_0265 – cysteine desulfurase	-1.8	Cofactors/vitamins
PurB – adenylosuccinate lyase	-1.8	Nucleotide metabolism
AccA - acetyl-CoA carboxylase carboxyltransferase	-1.7	Carbohydrate metabolism
Asd - aspartate-semialdehyde dehydrogenase	-1.7	Amino acid metabolism
Cjj81176_0443 - hypothetical	-1.7	Hypothetical
Cjj81176_0729 - hypothetical	-1.7	Hypothetical
Cjj81176_1348 – fibronectin binding protein	-1.7	Adhesin
FlpA – fibronectin binding protein	-1.7	Adhesin
LepA – translation elongation factor	-1.7	Translation
MogA - molybdenum cofactor biosynthesis protein	-1.7	Folding, sorting, degradation
PstB - phosphate transport ATP-binding protein	-1.7	Membrane transport
Pta – phosphate acetyltransferase	-1.7	Carbohydrate metabolism
PurA - adenylosuccinate synthetase	-1.7	Nucleotide metabolism
Adk – adenylate kinase	-1.6	Nucleotide metabolism
ArgG - Argininosuccinate synthase	-1.6	Amino acid metabolism
FtsH - ATP-dependent zinc metalloprotease	-1.6	Folding, sorting, degradation
GltA – citrate synthase	-1.6	Carbohydrate metabolism
LgtF - glycosyltransferase	-1.6	Glycan metabolism
SucD - succinyl coA-synthetase α chain	-1.6	Carbohydrate metabolism
WaaF – LOS heptosyltransferase	-1.6	Glycan metabolism
CheV	-1.5	Cell motility
CheY	-1.5	Cell motility

Cjj81176_0266 - hypothetical	-1.5	Hypothetical
Cjj81176_1062 - hypothetical	-1.5	Hypothetical
CoaE - dephospho-CoA kinase	-1.5	Cofactors/vitamins
DapA - dihydrodipicolinate synthase	-1.5	Amino acid metabolism
IlvE - branched-chain amino acid aminotransferase	-1.5	Amino acid metabolism

Total - 57
